# Prostatic-specific antigen (PSA) levels in patients with polycystic ovary syndrome (PCOS): a meta-analysis

**DOI:** 10.1186/s13048-019-0569-2

**Published:** 2019-10-15

**Authors:** Zeng-Hong Wu, Yun Tang, Xun Niu, Fei-Fei Pu, Xi-Yue Xiao, Wen Kong

**Affiliations:** 1 Department of Otolaryngology, Union Hospital, Tongji Medical College, Huazhong University of Science and Technology, Wuhan, Hubei China; 2 Department of Infectious Diseases, Union Hospital, Tongji Medical College, Huazhong University of Science and Technology, Wuhan, 430022 China; 3 Department of Critical Care Medicine, Union Hospital, Tongji Medical College, Huazhong University of Science and Technology, Wuhan, 430022 China; 4 Department of Orthopedics, Wuhan No.1 Hospital, Wuhan Integrated TCM & Western Medicine Hospital, Tongji Medical College, Huazhong University of Science and Technology, Wuhan, Hubei 430022 People’s Republic of China; 5 Department of Obstetrics and Gynecology, Union Hospital, Tongji Medical College, Huazhong University of Science and Technology, Wuhan, China; 6 Department of Endocrinology, Union Hospital, Tongji Medical College, Huazhong University of Science and Technology, Wuhan, 430022 China

**Keywords:** PCOS, Prostatic-specific antigen, Meta-analysis

## Abstract

**Purpose:**

The polycystic ovary syndrome (PCOS) is a reproductive endocrine disorder, clinically characterized by oligo-ovulation/chronic anovulation, menstrual irregularities, hyperandrogenism (such as hirsutism, acne), hyperinsulinemia, and obesity. Prostatic-specific antigen (PSA) has been identified as a potential new marker in PCOS women. Although the precise role of PSA in PCOS patients still remains undetermined, PSA might serve as a useful clinical marker and might even represent a new diagnostic criterion of hyperandrogenemia in females of PCOS.

**Methods:**

A meta-analysis was performed in the study to identify the association between the polycystic ovary syndrome and prostatic-specific antigen. To identify eligible original articles, we searched a range of computerized databases, including Medline via PubMed, EMBASE, CNKI and Web of Science with a systematic searching strategy. The characteristics of each study and standard mean differences (SMD) with corresponding confidence intervals (CIs) were calculated and subgroup analysis was performed to analyze heterogeneity.

**Results:**

A total of 532 patients from seven articles were included in the meta-analysis. We identified a significant relationship between polycystic ovary syndrome and prostatic-specific antigen, with a pooled SMD of 0.81 (95% CI: 0.58 to 1.04; *P* < 0.01). The pooled data were calculated with the random-effects model as a moderate significant heterogeneity was found among the studies.

**Conclusions:**

The meta-analysis suggested that there was a significant association between the polycystic ovary syndrome and prostatic-specific antigen and we should not ignore the role of PSA in the PCOS patients in clinical.

## Introduction

The polycystic ovary syndrome (PCOS) is a reproductive endocrine disorder, clinically characterized by oligo-ovulation/chronic anovulation, menstrual irregularities, hyperandrogenism (such as hirsutism, acne), hyperinsulinemia, and obesity [1]. PCOS affected approximately 5–10% in women of reproductive age [2, 3]. The real pathophysiological defect still remains unclear, but PCOS may be present with excessive androgen, with varying degrees of gonadotropin and metabolic abnormalities. PCOS remains a syndrome and no single diagnostic measure is insufficient for clinical diagnosis, while the biochemical markers of hyperandrogenemia appear to have highly variable diagnostic performance [4]. It was clearly noted that an extent of patients with PCOS exhibit overt abnormality in circulating androgens as PCOS patients demonstrate signs and symptoms of hyperandrogenism such as alopecia, acne, hirsutism, and ovulatory dysfunction [5, 6]. Androgen suppression after diagnosis of hyperandrogenism remains the primary basis for PCOS treatment in patients who do not wish to have immediate fertility [7]. Prostatic-specific antigen (PSA) is a serine protease and an essential marker widely used for the diagnosis of prostate cancer [8]. PSA not just produced by prostate gland but has been detected in some female tissues such as ovarian, endometrial tissues, breast, milk and amniotic fluid [9, 10]. PSA production seems to be associated by steroid hormones such as progestin, androgens and glucocorticoids. Zarghami et al. [11] has been clarified that PSA is up-regulated by androgens in females. PSA has been detected as a potential novel marker not only in PCOS women but also in hirsute women of hyperandrogenism [12, 13]. But in one study, it was reported that the PSA levels in serum were not valuable for the diagnostic of hirsutism [14]. Although the precise role of PSA in PCOS patients still remains undetermined, PSA might as for a helpful clinical marker and might even serve as a new diagnostic criterion of hyperandrogenemia in females of PCOS.

In the past decade, a growing number of papers have examined the PSA levels in the PCOS patients and try to investigate the associations between them, however, the results are controversial. In PCOS patients, some studies reported [7, 13] the level of circulating total PSA (tPSA) or free PSA (fPSA) is increased while some studies have found the opposite result [12]. Until now, there is no meta-analysis explored whether PCOS correlates with PSA. Thus, to further evaluate the link of serum PSA levels and PCOS, we conducted a meta-analysis of the literature on the subject to grade the strength of evidence. (Supporting information: PRISMA Checklist) [15].

## Materials and methods

### Search strategy

Studies detailing the polycystic ovary syndrome and total PSA or free PSA levels (mean ± SD) were distinguished for inclusion. In order to identify relevant original articles, we searched online open access computerized databases, including Medline, CNKI, EMBASE and Web of Science using the following words: ‘polycystic ovary syndrome’ ‘PCOS’ ‘prostatic specific antigen’ ‘PSA’ isolated by the Boolean operator OR or AND. Studies were searched up to August 2018 and no limits of language. We also reviewed the reference lists from the identified researches for further additional eligible publications. The titles and abstracts of the studies that could contain information regarding PCOS and serum PSA levels were assessed the full text. Two authors independently (and manually) reviewed all abstracts and screened the reference lists and disagreements were settled by consensus.

### Inclusion and exclusion criteria in our study

The inclusion criteria were: (1) all study and control subjects were limited to adults with PCOS diagnosed consistently by using either the consensus statement declared at Rotterdam [16] or National Institute of Health (NIH) [17] criteria; (2) no statistically significant difference to that in the PCOS group and control group in terms of body mass index (BMI) and age; (3) studies were restricted to humans, published in English, contained original data; (4) studies reported total PSA or free PSA, observational data were available; controls were women who did not have symptoms of PCOS; (5) studies in which all PCOS group members exclude any conceivable predisposing factor (such as Cushing syndrome, thyroid dysfunction, ovarian tumors) that may be related to their PCOS or were take the medicines (such as oral contraceptives, other hormonal therapies) at the time of the experimental. The exclusion criteria were: (1) duplicate publications, case reports, abstracts, non-English, review articles, editorials; (2) the information available was not sufficient for data analysis or extraction.

### Information extraction

Information was gathered for each study concerning name of first author, publication year, study design, sample size, analytical method used, and tPSA or fPSA levels, BMI, AHI (mean ± SD) in PCOS patients and non-PCOS subjects.

### Statistical analysis

We used the Newcastle-Ottawa Scale to assess the quality of each individual included study [18]. Meta-analysis was performed using Cochrane statistical software Review Manager 5.3. Heterogeneity was assessed by calculating the *I*^*2*^ index. An *I*^*2*^ value of 75 to 100% was as high level of heterogeneity, 50 to 75% as moderate heterogeneity level and an *I*^*2*^ value between 25 and 50% was considered to represent low heterogeneity level. An *I*^*2*^ value < 25% was considered homogeneous. If *I*^*2*^ value > 50% the random effects model (REM) was used to calculate standard mean differences (SMD) with 95% CI in PSA levels for each study and the pooled effect size and if *I*^*2*^ value < 50% the fix effects model (FEM) was used. Subgroup analysis used to determine the sources of heterogeneity. The sensitivity analysis was used to estimate the influence of each study in the meta-analysis by removing a different individual study each time.

## Results

### Search strategy result

**Fig. 1:**
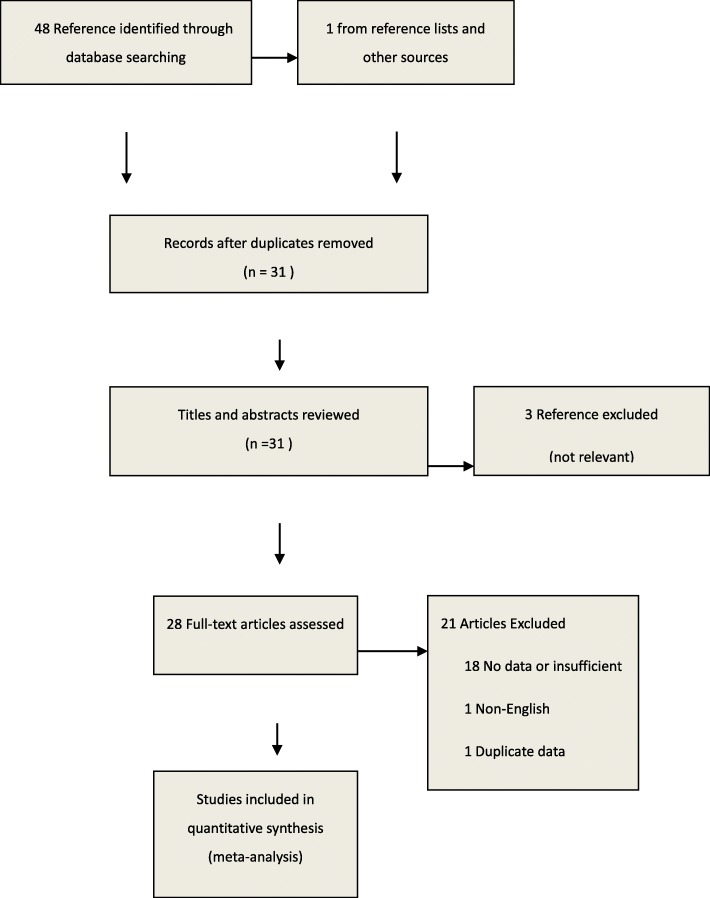
Search strategy to identify articles on the relationship between polycystic ovary syndrome and prostatic specific antigen levels

Forty-eight potentially relevant articles were identified from online databases and one from reference lists. We initially barring duplicates studies and 31 studies remained. Screening the titles and abstracts of these 31 articles drove us to exclude three articles that did not accord the inclusion criterion. Subsequent to reading the full content of the rest of the 28 articles as potentially regarding the link of PCOS and tPSA or fPSA levels, 18 were excluded as they did not report adequate information; one study was excluded as the outcome measure is improper; one study was duplicate and one study is non-English. Ultimately, seven eligible articles included 11 studies were fulfilled the eligibility criteria [7, 12, 19–23]. The selection process was appeared in Fig. 1 and the detailed information was listed in Table 1.

**Table 1:** Description of included studies

Source	Year	Country	Sample Size (PCOS/CG)	Study design	Mean BMI (kg/m^2^)		Mean Age, y	
					PCOS	CG	PCOS	CG
Bahceci et al. [19]^a^	2004	Turkey	30/30	Case-control	22.8 ± 5	21 ± 2.4	22.4 ± 3.9	22.7 ± 1.4
Bili et al. [20]	2014	Greece	43/40	Case-control	24.9 ± 5.9	22.7 ± 3.7	28.9 ± 5.0	30.8 ± 4.3
Gullu et al. [12]^a^	2003	Turkey	33/20	Case-control	26.04 ± 7.70	24.13 ± 6.67	23.04 ± 4.64	27.92 ± 7.81
Mardanian et al. [21]	2011	Iran	32/32	Case-control	24.78 ± 2.96	23.19 ± 2.24	26.38 ± 4.8	27.1 ± 4.9
Tokmak et al. [22]	2018	Turkey	42/47	Case-control	23.8 ± 4.6	20.8 ± 2.9	19 ± 2	18 ± 3
Ukinc A et al. [23]^a,b^	2009	Turkey	42/35	Case-control	26.35 ± 7.05	26.1 ± 6.9	23.29 ± 5.95	25.4 ± 5.0
Ukinc B et al. [23]^a,b^	2009	Turkey	20/35	Case-control	25.55 ± 5.29	26.1 ± 6.9	25.45 ± 9.36	25.4 ± 5.0
Vural et al. [7]	2007	Turkey	43/43	Case-control	23.45 ± 4.70	21.52 ± 3.01	21.4 ± 1.88	20.8 ± 2.28

Free androgen index (FAI) = total testosterone (TT)*100/sex hormone binding globulin (SHBG)

Data are expressed as mean ± SD

*Abbreviations*: *PCOS* Polycystic ovary syndrome, *CG* Control group, *NG* Not given, *BMI* Body mass index, *PSA* Prostatic specific antigen, *LH* Luteinizing hormone, *FSH* Follicle stimulating hormone

^a^Data included total PSA and free PSA

^b^Data included two subgroups PCOS patients: anovulatory PCOS (Group A) and ovulatory PCOS (Group B)

The meta-analysis involving a total sample size of 532 (285 cases and 247 controls) and all of them were case–controls studies. Three studies [12, 19, 23] investigated total PSA and free PSA levels and one study [23] reported anovulatory PCOS group and ovulatory PCOS group patients. Six researches [7, 12, 19, 20, 22, 23] investigated European populations and one [21] analyzed Asian populations. Four studies [12, 19, 22, 23] were measured the free testosterone and three [7, 21, 23] reported LH/FSH ratio, two [19, 20] were reported the free androgen index (FAI).

### Meta-analysis results

**Fig. 2:**
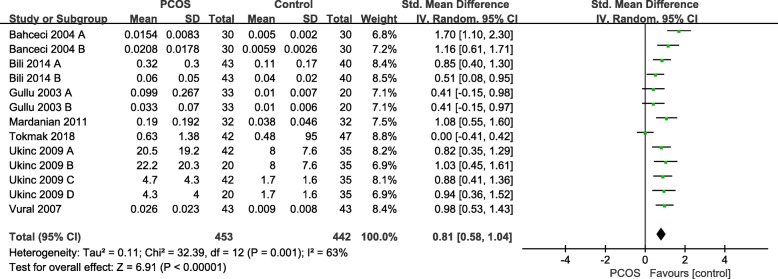
Relationship between PCOS with serum PSA levels. Calculation based on random effects model. Results are expressed as standard mean difference (SMD) and 95% confidence intervals (95% CI). Bahceci and Gullu’s study included total PSA (Group A) and free PSA (Group B); Ukinc’s study included two subgroups PCOS patients: anovulatory PCOS and ovulatory PCOS patients (total PSA: Group A vs Group B; free OSA: Group C vs Group D)

The forest plot for the link of PCOS with serum PSA levels was shown in Fig. 2. The results showed a significant connection between PCOS and serum PSA levels, with a pooled SMD of 0.81 (95% CI: 0.58 to 1.04; *P* < 0.01; REM).

### Subgroup analysis

#### PSA

**Total PSA** the results were significant, with a corresponding value of 0.84 (95% CI: 0.49 to 1.19, *P* < 0.01) under the REM. **Free PSA** the results were significant, with a corresponding value of 0.76 (95% CI: 0.53 to 0.99, *P* < 0.01) under the FEM Table 2.

**Table 2:** Results of subgroup analysis among PCOS vs controls

Subgroup	Studies Included (N)	Sample size (PCOS/CG)	Chi square (*df*)	*P* value	Pooled Overall SMD (95% CI)	Heterogeneity (*I*^*2*^)
Total PSA	8	285/247	26.98 (7)	.001	0.84 (0.49–1.19)	74
Free PSA	5	168/125	5.38 (4)	.001	0.76 (0.53–0.99)	26
BMI ≥ 25	6	190/110	4.50 (5)	.001	0.76 (0.54–0.97)	0
BMI < 25	7	263/262	27.83 (6)	.001	0.87 (0.48–1.26)	78
Age ≥ 25	5	158/182	3.43 (4)	.001	0.84 (0.62–1.07)	0
Age < 25	8	295/260	28.45 (7)	.001	0.78 (0.42–1.14)	75
Free testosterone	9	292/217	28.78 (8)	.001	0.80 (0.47–1.13)	72
LH/FSH	6	199/145	0.68 (5)	.001	0.95 (0.74–1.15)	0
FAI	4	146/140	10.65 (3)	.001	1.02 (0.55–1.50)	72

#### Mean BMI in PCOS

**BMI ≥ 25** the total SMD in the studies with average BMI ≥ 25 was significant, with a corresponding value of 0.76 (95% CI: 0.54 to 0.97, *P* < 0.01). **BMI < 25** the total SMD in the studies with average BMI < 25 was significant, with a corresponding value of 0.87 (95% CI: 0.48 to 1.26, *P* < 0.01) Table 2.

#### Mean age in PCOS

**Age ≥ 25** the total WMD in the studies with average age ≥ 25 was significant, with a corresponding value of 0.84 (95% CI: 0.62 to 1.07, *P* < 0.01). **Age < 25** the total WMD in the studies with average age < 25 was significant, with a corresponding value of 0.78 (95% CI: 0.42 to 1.14, *P* < 0.01) Table 2.

#### Laboratory measurement

**Free testosterone** the total WMD in the studies with Free testosterone was significant, with a corresponding value of 0.8 (95% CI: 0.47 to 1.13, *P* < 0.01). **LH/FSH** the total WMD in the studies with LH/FSH was significant, with a corresponding value of 0.95 (95% CI: 0.74 to 1.15, *P* < 0.01). **FAI** the total WMD in the studies with LH/FSH was significant, with a corresponding value of 1.02 (95% CI: 0.55 to 1.50, *P* < 0.01) Table 2.

### Sensitivity analysis and quality assessment

The coupled forest plots show moderate heterogeneity (*I*^*2*^ = 63%) and when we removed Tokmak’s study the results dramatically influenced the pooled results (*I*^*2*^ decreased from 63 to 37%) in the meta-analysis under the REM. The results of sensitivity analysis showed that the pooled value ranged from 0.78 (95% CI: 0.54 to 1.02) to 0.88 (95% CI: 0.70 to 1.07). Moreover, in subgroup analyses (*I*^*2*^ = 74% for total PSA; *I*^*2*^ = 26% for free PSA; *I*^*2*^ = 0% for BMI ≥ 25; *I*^*2*^ = 78% for BMI < 25; *I*^*2*^ = 0% for age ≥ 25; *I*^*2*^ = 75% for age < 25; *I*^*2*^ = 72% for free testosterone; *I*^*2*^ = 0% for LH/FSH; *I*^*2*^ = 72% for FAI) also analysis. However, in free PSA subgroup, the results dramatically influenced by removed Bahceci’s group B study (*I*^*2*^ decreased from 26 to 0%). Table 3 summarizes the results of the quality assessment. The methodological quality was rated as high in six study, moderate in one study.

**Table 3:** Methodological assessment according to the Newcastle–Ottawa scale

Study	Selection				Comparability	Outcome			Total score^a^
	Representativeness	Selection	Ascertainment	Outcome of interest		Assessment	FU	Adequacy of FU	
Bahceci et al. [19]	*	*	*	*	**	*	*	*	9
Bili et al. [20]	*	*	*	*	*	*	*	*	8
Gullu et al. [12]	*	*	*	*	*	*	*	*	8
Mardanian et al. [21]	*	*	*	*	**	*	*	–	8
Tokmak et al. [22]	*	*	*	*	**	*	*	*	9
Ukinc A et al. [23]	*	*	*	*	*	*	*	*	8
Ukinc B et al. [23]	*	*	*	*	*	*	*	*	8
Vural et al. [7]	–	*	*	*	*	*	*	*	7

– indicates no stars

*FU* Follow-up

^a^We considered a study to be of high quality when the total score was eight or nine stars, moderate quality when the total score was six or seven stars, and low quality when the total score was five stars or fewer

## Discussion

This meta-analysis suggested that PCOS was significantly linked to serum tPSA or fPSA levels (SMD = 0.81, 95% CI: 0.58 to 1.04; *P* < 0.01) and in PCOS patients the serum PSA was increased when compared with controls. Although, the value of *I*^*2*^ = 63% (*I*^*2*^ > 50, *P* < 0.01), indicating that there existed moderate heterogeneity so we performed the subgroup analysis to found the sources of heterogeneity so the meta-analysis results of our study could serve as the relationship between PCOS and serum PSA levels in PCOS patients. Furthermore, sensitivity analysis exhibited that after any individual study was omitted or when REM was converted to FEM, the overall results and conclusion still held.

Hyperandrogenemia is already a well-known feature of PCOS. Although the source of serum PSA in women is not clear yet, it might reflect androgen action in one or more androgen- sensitive tissues and might also be a reliable biochemical marker of the biological action of androgen [13]. It has been recently reported in female colorectal and breast cancer; the results suggest that serum PSA might play a role in the diagnosis of these cancers [24, 25]. Futhermore, serum PSA levels are increased in hirsute patients and accompanying the degree of hyperandrogenism [26–28]. However, the exact mechanism of serum PSA participation in PCOS, still remains to be elucidated because the lake of enough related pathophysiology studies. Mardanian et al. [21] found positive correlation between tPSA and fPSA with DHEAS and hirsutism and LH/FSH ratio. Similarly, Vural et al. [7] found a positive correlation between tPSA and DHEAS, total testosterone and a negative correlation between tPSA and SHBG. While Rudnicka et al. [29] did not find the correlation between tPSA and DHEAS and SHBG. Ukinc et al. [23] found there were no significant differences between ovulatory and noovulatory PCOS women in respect to PSA concentrations, so in our meta-analysis we included both the study subjects. Obiezu et al. [30] demonstrated that urinary PSA and human glandular kallikrein 2 were significantly higher in patients with PCOS than in healthy subjects. Burelli et al. [31] did not observed significant differences in PSA serum during various stages of the menstrual cycle, or between pre- and postmenopausal women and suggests that PSA production is not affected by hormonal changes during the menstrual cycle, and that the source of its production is not within the female reproductive system. In our meta-analysis, due to the lake of enough studies we cannot conclude the PSA serum is affected by hormonal during various stages of the menstrual cycle. Previous studies have documented the presence of active androgen response elements within the PSA enhancer regions and gene promoter based on the truth that is up-regulated by androgens and hypothesis that serum PSA may be a novel biomarker of hyperandrogenism in females [32, 33]. But the pathogenesis of serum PSA in PCOS is not clearly understood. Our meta-analysis result is consistent with the results from most previous studies as the serum PSA was increased compared with controls in PCOS patients with the summary SMD was 0.81 (95% CI: 0.58 to 1.04; *P* < 0.01) under the REM.

A few studies have determined the diagnostic value of PSA and fPSA in women with PCOS. Ukinc et al. [7] found that the fPSA is present with equilibrium with PSA in serum and also found the diagnostic value of fPSA reached 70.5% sensitivity and 82% specificity for PCOS, more importantly, the author also recommends that PSA could be used for diagnosis of PCOS with high sensitivity, specificity, and diagnostic accuracy. Interestingly, Mardanian et al. [21] study the diagnostic value of PSA and reported PSA level to provide sensitivity and specificity of 85 and 80%, respectively in women with anovulatory PCOS. Another study reported tPSA and tPSA:fPSA ratio have similar diagnostic performance in women with PCOS [20]. Agreeing with these authors, in the subgroup analysis of our meta-analysis we found both tPSA and fPSA increased in PCOS women when compared with controls. Few studies have focused on the level of PSA in women with BMI. Circulating PSA level is suggest to be related to the obesity when BMI was found to be higher in hirsute women with PCOS [7]. Althought, in our BMI ≥ 25 and BMI < 25 subgroup analysis the results were significant, with a corresponding value of 0.76 (95% CI: 0.54 to 0.97, *P* < 0.01) and 0.87 (95% CI: 0.48 to 1.26, *P* < 0.01) respectively. But more researches are needed to conduct to find the relationship between serum PSA levels and obesity or BMI in PCOS patients. Some authors noted that there was no direct correlation between PSA levels and age [34]. In terms of age subgroup analysis, there was no obvious difference between age ≥ 25 and age < 25, but more researches s are required to evaluate if serum PSA levels are influenced by age.

The total testosterone (TT) is often used to evaluated androgen levels in PCOS patients in clinical. However, in the blood circulation, a part of TT exists in a free form, one part can transiently bind to albumin, and the other part binds to SHBG and only the first two have the physiological activity of androgen and testosterone which binds to SHBA does not [35]. Therefore, some studies [36, 37] points out that the diagnostic criteria based on free testosterone and FAI only consider physiologically active androgen levels, excluding the effects of testosterone levels combined with SHBG, and are superior to the methods for measuring TT concentration in evaluating hyperandrogenemia. Corresponding, in our laboratory measurement subgroup analysis, there was no obvious difference between free testosterone, LH/FSH and FAI subgroup.

There was no consistent conclusion until now about the association of PCOS with serum PSA levels and this is the first meta-analysis to approve this mechanism in PCOS patients. But some potential limitations of the meta-analysis should be taken into consideration when interpreting the results of our study. First, due to sparse data bias, in our subgroup analysis on the different variables could not undertake since there were insufficient data and limited studies. Second, the sample size was relatively small and may affect the accuracy of our results and many large-scale studies should be performed to convince it. Third, different assay methodology or instruments to detected serum PSA levels may also bias the results as well as the uniformity PCOS diagnostic criteria. In additional, hyperandrogenism plays an important role in the pathogenesis of PCOS women and may influence the progression and development of PCOS, but we were not able to assess this potential impact. Finally, owing to the difficulty in getting the abstracts or full texts of articles published in other languages, we only included studies in English. So, our results should be interpreted with caution and need more further researches.

## Conclusion

Although our meta-analysis showed the increased tPSA or fPSA levels in patients with PCOS, while the pathophysiology studies should be further investigated. Moreover, it can be concluded that PSA could be used as a novel marker for PCOS women because both tPSA and fPSA levels were found to be significantly higher in patients with PCOS than in healthy subjects.

## Data Availability

Not applicable.

## Abbreviations

FEM: Fix effects model
PCOS: The polycystic ovary syndrome
PSA: Prostatic-specific antigen
REM: Random effects model
SMD: Standard mean differences
